# Draft genome for *Pseudomonas alkylphenolica* IMGN1, isolated from soil

**DOI:** 10.1128/mra.00457-24

**Published:** 2024-07-31

**Authors:** Jaeyeon Lee, Bon-Jin Ku, Yeongjun Kim, Jeong A. Han, Eun Yu Kim, Ho-Seok Lee

**Affiliations:** 1 Center for Genome Engineering, Institute for Basic Science, Daejeon, South Korea; 2 Department of Biology, College of Science, Kyung Hee University, Seoul, South Korea; 3 Gyeonggido Agricultural Research and Extension Services, Hwaseong, South Korea; 4 Division of Natural and Applied Sciences, Duke Kunshan University, Kunshan, China; 5 Environment Research Center, Duke Kunshan University, Kunshan, Jiangsu, China; The University of Arizona, Tucson, Arizona, USA

**Keywords:** *Pseudomonas*, *Pseudomonas alkylphenolica*, biocontrol, biocontrol agent

## Abstract

Biocontrol using organisms like *Pseudomonas alkylphenolica* offers a viable alternative to chemical pesticides, enhancing plant growth and reducing environmental impact. This study details the genome of *Pseudomonas alkylphenolica* IMGN1, a strain known for promoting plant growth, advancing our understanding of biocontrol mechanisms.

## ANNOUNCEMENT

IMGN1 belongs to *Pseudomonas alkylphenolica*, a genus of the Gammaproteobacteria class. *Pseudomonas* comprises rod-shaped, Gram-negative bacteria widely found in water and plant seeds (1). This bacterial group is recognized for its production of diverse antifungal compounds, which are directly associated with biocontrol activity (2). Specifically, IMGN1, a strain of *Pseudomonas alkylphenolica*, is acknowledged as a plant growth-promoting bacterium by enhancing the efficiency of phosphorus utilization in plants. This announcement will provide genomic resources for comparative studies, facilitating the broader utilization of *Pseudomonas alkylphenolica*.

IMGN1 was isolated from a depth of 15 cm in soil collected from Yangpyeong province (37.5970422′N, 127.5983547′E), Republic of Korea, in 2018. It was spread onto a tryptic soy agar plate incubated at 15°C for 16 hours to obtain a pure colony.

The extraction of genomic DNA from a single colony was performed using Maxwell RSC Tissue DNA kit. The obtained genomic DNA was divided into two portions in order to be subjected to different sequencing methods. Initially, a segment was cut using the Megaruptor and then purified using AMPure PB magnetic beads to select a specific size. The PacBio sequencing library was generated using the PacBio SMRTbell Prep Kit v.3.0. Following that, HiFi sequencing was conducted using the PacBio Sequel II, resulting in 119,660 reads with a mean depth of 207.5. The average length of HiFi reads was 9,835 bp, with an N50 value of 10,509 bp.

The remaining portion was utilized to create the Illumina sequencing library using the TruSeq DNA Nano Library Prep Kit from Illumina, Inc. Following that, sequencing was conducted on the Illumina HiSeq X Ten platform using a 2 × 150 paired-end protocol, resulting in 7,409,706 reads. In addition, Trimmomatic v.0.38 was used to perform quality filtering and adaptor trimming, with the requirement that 90% of the bases have a Phred score of 30 or higher (3).

The draft genome assembly was generated by assembling the HiFi reads *de novo* using HGAP v.4 (4). Afterward, the genome was refined using Illumina reads, and Pilon v.1.21 was employed for three rounds of polishing. The complete genome of IMGN1 was composed of one contig, amounting to a total of 5,665,012 bp. It had an average GC content of 61.0% and an N50 value of 5,665,012 (5). The assembly quality was 99.19%, evaluated using BUSCO v.5.1.3 (6).

The NCBI Prokaryotic Genome Annotation Pipeline (PGAP) was used to predict genes, resulting in 4,969 coding sequences, 14 tRNA genes, and 24 rRNA genes (7). The compiled draft genome was cross-referenced with the Genome Taxonomy Database (GTDB) using GTDB-Tk v.2.3.2 and classified as *Pseudomonas alkylphenolica*. It was cross-referenced with GTDB using GTDB-Tk v.2.3.2 (8). Default parameters were utilized in every step, unless specified otherwise.

The genetic makeup includes 5,018 predicted proteins, encompassing genes implicated in a spectrum of essential cellular processes, including transcription, membrane, and envelope biogenesis, alongside translation, ribosomal structure, and biogenesis. Relative to its closely related counterparts, this genome demonstrates a notably higher GC content (9).

## Data Availability

The annotated draft genome sequence has been submitted to the National Center for Biotechnology Information and assigned the GenBank accession number CP152293. The BioProject database accession number is PRJNA1092547, and the BioSample accession number is SAMN40626785. The Sequence Read Archive information is available under the accession numbers SRX24204479 and SRX24204780.

## AFTER EPUB

*[This article was published on 31 July 2024 with an incorrect accession number. The accession number was corrected in the current version, posted on 7 August 2024.]*
